# Getting a head in hard soils: Convergent skull evolution and divergent allometric patterns explain shape variation in a highly diverse genus of pocket gophers (*Thomomys*)

**DOI:** 10.1186/s12862-016-0782-1

**Published:** 2016-10-10

**Authors:** Ariel E. Marcy, Elizabeth A. Hadly, Emma Sherratt, Kathleen Garland, Vera Weisbecker

**Affiliations:** 1 School of Biological Sciences, University of Queensland, St. Lucia, QLD 4072 Australia; 2 Department of Biology, Stanford University, Stanford, 94305-5020 CA USA; 3 Department of Evolution, Ecology and Genetics, Research School of Biology, The Australian National University, Canberra, 2601 ACT Australia

**Keywords:** Environmental selection pressure, Evolutionary development, Heterochrony, Incisor procumbency, Parallel evolution, Principal component analysis, Repeated evolution, Subterranean niche

## Abstract

**Background:**

High morphological diversity can occur in closely related animals when selection favors morphologies that are subject to intrinsic biological constraints. A good example is subterranean rodents of the genus *Thomomys*, one of the most taxonomically and morphologically diverse mammalian genera. Highly procumbent, tooth-digging rodent skull shapes are often geometric consequences of increased body size. Indeed, larger-bodied *Thomomys* species tend to inhabit harder soils. We used geometric morphometric analyses to investigate the interplay between soil hardness (the main extrinsic selection pressure on fossorial mammals) and allometry (i.e. shape change due to size change; generally considered the main intrinsic factor) on crania and humeri in this fast-evolving mammalian clade.

**Results:**

Larger *Thomomys* species/subspecies tend to have more procumbent cranial shapes with some exceptions, including a small-bodied species inhabiting hard soils. Counter to earlier suggestions, cranial shape within *Thomomys* does not follow a genus-wide allometric pattern as even regional subpopulations differ in allometric slopes. In contrast, humeral shape varies less with body size and with soil hardness. Soft-soil taxa have larger humeral muscle attachment sites but retain an orthodont (non-procumbent) cranial morphology. In intermediate soils, two pairs of sister taxa diverge through differential modifications on either the humerus or the cranium. In the hardest soils, both humeral and cranial morphology are derived through large muscle attachment sites and a high degree of procumbency.

**Conclusions:**

Our results show that conflict between morphological function and intrinsic allometric patterning can quickly and differentially alter the rodent skeleton, especially the skull. In addition, we found a new case of convergent evolution of incisor procumbency among large-, medium-, and small-sized species inhabiting hard soils. This occurs through different combinations of allometric and non-allometric changes, contributing to shape diversity within the genus. The strong influence of allometry on cranial shape appears to confirm suggestions that developmental change underlies mammalian cranial shape divergences, but this requires confirmation from ontogenetic studies. Our findings illustrate how a variety of intrinsic processes, resulting in species-level convergence, could sustain a genus-level range across a variety of extrinsic environments. This might represent a mechanism for observations of genus-level niche conservation despite species extinctions in mammals.

**Electronic supplementary material:**

The online version of this article (doi:10.1186/s12862-016-0782-1) contains supplementary material, which is available to authorized users.

## Background

Animal populations modify their existing anatomy in response to selection. Functional morphology is therefore a compromise between adaptive forms and possible forms [1] given the organism’s evolutionary history [2]. The range of possible adaptive forms is also dictated by what morphological changes can be produced by intrinsic processes such as development or allometry) [3, 4]. In particular, allometry—the disproportionate shape change of a trait due to a change in body size—plays a key role in shaping the evolution of new forms [3]. However, because of limitations imposed by the available morphospace, phylogenetic constraints, and evolutionary time [5], these intrinsic processes can reduce the range of adaptations to new selection pressures. The impact of conflicts between morphological constraints and functional selection can be observed across phylogenetic scales, ranging from at the population level (e.g. [6, 7]) and as large as the three major mammalian subclasses (e.g. [8, 9]).

**Fig. 1:**
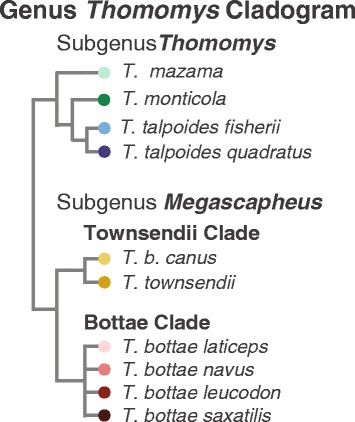
Genus *Thomomys* cladogram. Cladogram of taxa in the Northern California study region (adapted from [20, 21]). Note that *T. (M.) bottae canus*, despite its species name, is more closely related to *T. (M.) townsendii* in the *T. M.* Townsendii clade than to the *T. M.* Bottae clade taxa. Note that the *T. M.* Bottae clade is presented as a soft polytomy, and support for internal nodes in the subgenus *Thomomys* is very low [20]

Fossorial mammals are often used as model organisms to understand the evolutionary interaction between the extrinsic environmental pressures and the intrinsic processes that generate the variation on which natural selection acts [10–15]. Fossoriality is well known to represent an immense selection pressure on the mammalian skeleton [12, 16]. Digging requires 360–3400 times more energy per-distance than walking [17, 18]. The remarkably species-rich fossorial western pocket gophers (genus *Thomomys*) in northern California [19] are thus a good choice to investigate diverse adaptations to digging. The clade also has a well-established species-level phylogeny (Fig. 1) [20, 21]. In a relatively small geographic area, two subgenera (*T. Thomomys* and *T. Megascapheus*) have radiated into 10 taxa (5 species containing 7 subspecies; Fig. 1) that contend with varying soil and climate conditions at the confluence of coastal, montane, and desert basin regions [20, 22]. Unlike most other animal genera, whose species tend to occupy very similar environments [2], *Thomomys* pocket gophers provide an opportunity to investigate morphological responses of closely related taxa to great variation in external conditions.

In response to the selection pressures of fossoriality, two regions of the pocket gopher skeleton in particular appear adapted to digging: powerful forearms reflect extensive claw-digging, and—often in regions of hard soil—skull modifications allow for prolonged tooth-digging [16]. However, different taxa vary along this claw- to tooth-digging spectrum [23]. Body size is also a major factor in the evolution of pocket gophers, as there appears to be intense selection to reduce burrow dimensions as much as body size allows: this minimizes the volume of soil moved and the cross-section sheared while foraging underground [18, 24–26]. The exact energetic cost of digging thus depends on a species’ body size, specific digging adaptations, as well as the local soil type [17, 18]. As *Thomomys* gophers are territorial dietary generalists, competition seems to distribute the different species into neighboring, mostly non-overlapping ranges [22, 27]. These range boundaries correspond with changes in soil, suggesting that interspecific differences in body size and digging adaptations confer competitive dominance to one species over another through maximizing foraging efficiency in the local soil [17, 22, 23, 25, 27].

Body size and tooth-digging present a potential morphological trade-off that may underlie the apparent shifts in competitive dominance of one species over its neighbors corresponding with subtle soil changes across very short distances—as little as a few meters [22]. Sustained digging with teeth, the hardest structures in the vertebrate body, requires a derived skull morphology of procumbent incisors (incisor tips with an anterior projection greater than 90° relative to the rostral plane) [16]. Several studies show that procumbent species use less energy in harder soils and/or have higher burrowing rates than their “orthodont” counterparts (i.e. those with the ancestral condition [28] of acutely angled incisors) [17, 23, 29–31]. Increasing body size is associated with greater muscular strength and with an increased incisor angle [23]. An increase in rostral length, resulting in a larger incisor arc radius, appears to underlie this allometric mechanism [32, 33]. This seems to explain why harder soils are generally inhabited by larger species. In contrast, a large body size may be disadvantageous in softer, sandier, lower-clay soils because larger taxa must move a greater soil volume to create larger, less stable burrows [18].

In addition to allometry, wholesale craniodental rearrangements have also been implicated in the evolution of tooth-digging, and may provide a mechanism for increasing incisor procumbency without associated increases in body size [28, 33]. Here, the posterior movement of the incisor root position creates a more obtuse angle with minimal changes in the shape or length of the rostrum [28, 33]. This intrinsic process represents a key innovation in pocket gophers, having evolved at least three times in subgenus *T. Megascapheus* alone [28].

Forelimb adaptation is also expected to play a role in the evolution of gopher morphology. All *Thomomys* gophers claw-dig—even procumbent species preferentially use claw-digging when soil compaction does not require tooth-digging [23]. Therefore, procumbent tooth-digging species have more than one digging mode, compared to the ancestral orthodont species which must rely on claw-digging (aside from very limited employment of teeth to remove plant roots) [16]. It is therefore expected that orthodont species would have more derived limb long bones compared to tooth-digging species. In contrast to the mandible, which exhibits plasticity in response to harder foods [34], recent research on muscle attachment sites suggests that long bone shape is mostly determined through inheritance [35–37].

Overall, species within the genus *Thomomys* tend to conform to the expectation that body size and soil type are tightly linked [22]: subgenus *T. Thomomys* retains the ancestral condition [28] of a small body size and tends to inhabit softer soils [22], while the larger subgenus *T. Megascapheus* inhabits harder soils [22, 27, 28]. Females of the largest species, *T. (Megascapheus) townsendii* (245 g) weigh about four times those of the smallest species, *T. (Thomomys) talpoides* (64 g) [38, 39]. The larger subgenus, *T. Megascapheus*, also tends to have more procumbent incisors, suggesting a role for allometry in the evolution of this cranial morphology [28]. Unexpectedly, the smallest taxa (two *T. (T.) talpoides* subspecies) break the genus-wide trend by inhabiting hard soils [27]. This exception could be due to digging adaptations arising from a more complex evolutionary mechanism than allometry alone. This suggests that pocket gophers adapt to fossoriality through a variety of intrinsic mechanisms, which interact differentially in taxa experiencing different selection pressures exerted by different soil types.

**Fig. 2:**
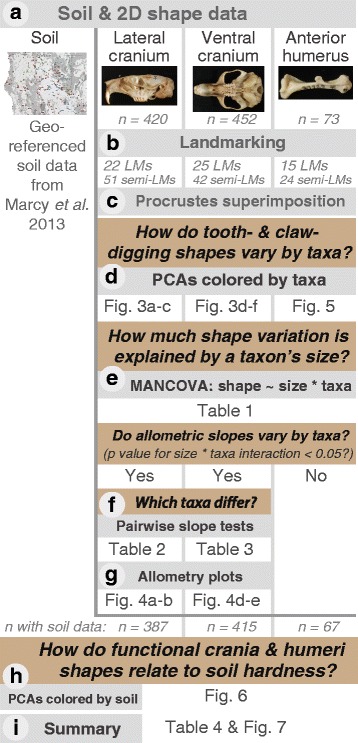
Methods and analyses summary. Soil data included three indices of conditions contributing to soil hardness: percent clay, bulk density, and linear extensibility (i.e. how much soil hardens when moisture is low); 2D photographs were taken of each specimen **a**. Landmarks (LMs) and semilandmarks (semi-LMs) were used to capture both homologous points and curves, respectively across the different taxa **b**. All of our statistical analyses were performed in the R environment using the geometric morphometric package, *geomorph* **c**–**h**. The last figure and table present an interpretative summary of the shape with soil type **i**

Here, we use the fine-grained taxonomic structure and geographic distribution of northern Californian pocket gophers to assess how environment, constraint, and intrinsic shape patterning processes may produce a diverse range of morphologies among closely related mammals. First, we test for shape divergence between the 2 subgenera, 3 clades, and 10 distinct taxa (species or subspecies; see Fig. 1) in cranial and humeral shapes using principal components analyses. Second, we investigate the variation in the impact of allometry on shape using MANCOVAs, allometry plots, and pairwise tests of homogeneity of slopes. Third, we visualize the association of shape with three soil conditions—previously shown to separate gophers into their respective ranges [27]—on cranial and humeral shape. Finally, we assess the evidence for convergent evolution within the genus by comparing forelimb and skull morphologies between more distantly related taxa inhabiting similar soils (Fig. 2).

## Methods

### Samples

We examined 452 crania and 73 humeri from adult female specimens of genus *Thomomys* pocket gophers housed in the Museum of Vertebrate Zoology (MVZ) at the University of California, Berkeley in the United States (for sample sizes per analysis, see Fig. 2 and for samples sizes by taxa, see Additional file 1: Table S1). Because male pocket gophers continue to grow past sexual maturity, we included only female specimens to remove ontogeny-related allometry [40]. Female specimens were included only if they were past sexual maturity based on the degree of suture closure (after [40]). MVZ specimen catalog numbers are given in Additional file 2: Table S1.

At least 25 crania represented each of the 10 distinct taxa present (Fig. 1): 4 taxa from subgenus *T. Thomomys*: *T. (Thomomys) mazama*, *T. (T.) monticola, T. (T.) talpoides fisherii*, and *T. (T.) talpoides quadratus*; 2 taxa from the subgenus *T. Megascapheus* Townsendii clade: *T. (Megascapheus) bottae canus* and *T. (M.) townsendii nevadensis*; and 4 taxa from the subgenus *T. Megascapheus* Bottae clade: *T. (M.) bottae laticeps, T. (M.) bottae navus, T. (M.) bottae leucodon,* and *T. (M.) bottae saxatilis*. Three taxa, *T. (T.) mazama, T. (T.) monticola*, and *T. (M.) bottae navus*, had sufficient sampling to compare the shape variation between regional subpopulations (Additional file 3: Table S3). We included at least 20 humeri from each of the 3 major clades within *Thomomys* in the study area (Additional file 1: Table S1). For brevity, we refer to the 10 sampled distinct species or subspecies as “taxa” throughout.

### Soil data

Soil data associated with each specimen were taken from Marcy et al. (2013) [27]. Geo-referenced soil data combines genus *Thomomys* locality data from Arctos and soil data from the U.S. Department of Agriculture, National Resources Conservation Service (NRCS) STATSGO 2006 Digital General Soil Map of the United States [27]. The dataset for this study included values for percent clay, bulk density (oven dry at 1/3 bar water tension), and linear extensibility—each reported as a weighted average aggregation of the conditions found between the soil surface to a depth of 20 cm.

### Geometric morphometrics

Cranial and humeral shape were captured from digital photographs in two views each: crania were photographed in ventral and lateral views, and humeri in anterior and lateral views. We followed the Grinnell Resurvey Project’s protocol for photography [41] (see also Additional file 4: Supplementary methods). Crania were photographed according to protocols by Fernandes et al. (2009) [48]. Humeri were photographed according to protocols by Steiner-Souza et al. (2010) [42]. A ruler adjusted to be level with the photographic plane provided a standardized scale during image processing.

Cranial and humeral shapes were characterized using 2D landmarks and semilandmarks (n numbers listed in Fig. 2b; shown on specimen photos in Additional file 5: Figure S1 with definitions given in Additional file 6: Table S4), digitized in tpsDIG (v 2.17) [43]. To reduce acquisition error, only one of us (AEM) obtained photographs and landmarks. Photographs were randomly sorted prior to digitizing to eliminate systematic error. We assessed operator error using 20 photographs of one specimen taken throughout the data acquisition process for 4 individuals representing each of the clades and the range of body sizes in our sample (after [44]). The mean estimated measurement error based on centroid size variance averaged 0.05 %. Photographs of specimens with evidence of arthritis, broken incisors, or other damaged elements were removed (7.6 % of the original dataset).

For each cranial and humeral dataset, the landmark coordinates were aligned using a generalized Procrustes superimposition implemented in the R package *geomorph* (v. 3.0) [45, 46]. Superimposition of each set of landmark coordinates removes differences in size, position, and orientation, leaving only shape variation [47]; semilandmarks were permitted to slide along their tangent directions in order to minimize Procrustes distance between specimens [48]. The resulting Procrustes tangent coordinates for each view were used as shape variables in all subsequent analyses (Fig. 2c).

### Statistical analyses

**Fig. 3:**
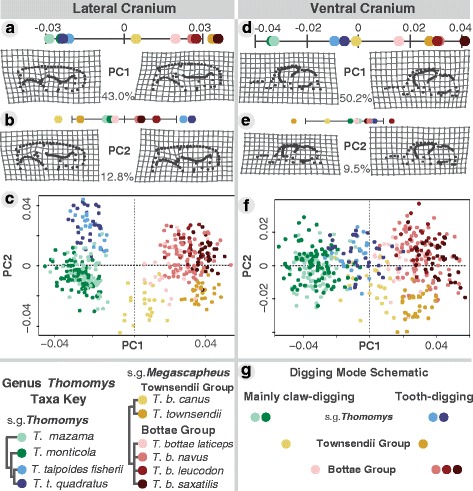
Cranial shape principal component analysis. For all cranial PCs, positive scores correspond with better tooth-digging shapes **a**–**g**. Lateral cranial principal component (PC) 1 captures incisor procumbency due to allometry, points for each taxa give their average value along the PC axis **a** while PC2 appears to capture incisor procumbency related to a shift in incisor root position **b**. Lateral cranial PCA morphospace for each individual in the dataset **c**. Ventral cranium view PC1 captures skull robustness **d** while PC2 differentiates subgenus *Megascapheus* based on muscle attachment sites on the zygomatic arch as well as the orientation of the foramen magnum **e**. Ventral cranial PCA morphospace **f**. Digging mode schematic presents the relative use of tooth- and claw-digging used by taxa in our study region based on the literature and inferences from our shape analyses **g**

All of our statistical analyses were performed in R (v3.2.3) [49] using the R package *geomorph* (v. 3.0) [45, 46]. We performed a principal component analysis (PCA) on the shape variables of each dataset to visualize the variation among individuals (Fig. 2d). To interpret the shape variation described by each PC axis, we used thin-plate spline deformation grids [50], which illustrate the shape change from the mean shape to the minima and maxima of each PC axis (e.g. see Fig. 3).

We estimated the allometric relationships between cranial shape and size, and humeral shape and size, by running multivariate analysis of covariance (MANCOVA) models using log-transformed centroid size, taxa affiliation, and their interaction as model effects (Fig. 2e). Statistical significance of each analysis was evaluated using Goddall’s (1991) [51] F-ratio and a randomized residual permutation procedure using 1000 iterations [52]. If the interaction terms were significant, this indicated that the static allometric trajectories (i.e. “slopes”—the shape change in multivariate space associated with size variation in adult centroid sizes) differed between taxa. When this was the case (in the cranial views, see Fig. 2e), we performed a pairwise test for homogeneity of slopes using the “advanced.procD.lm” function in *geomorph* (Fig. 2f). These tests identified which taxa significantly differed in allometric slope from each other. In these analyses, the pairwise angles between taxa-specific slopes were calculated in degrees and assessed for similarity through permutation using 1000 iterations. Taxa-specific slopes were visualized using plots of the first principal component of the predicted shape scores from the multivariate regression against log-transformed centroid size (after [53]) (Fig. 2g). Patterns of static allometry derived from each view’s MANCOVA were also visualized using the regression score (after [54]).

To visualize how shape variations in the cranial and humeral views correspond to local soil conditions, specimen points in the cranial and humeral PCAs were colored according to the percent clay, bulk density, and linear extensibility they were found in (data from Marcy et al. 2013 [27]) (Fig. 2h).

## Results

### Variation in cranial shape

Principal component analyses (PCA) (n_lateral_ = 452; n_ventral_ = 420) revealed that the first two principal component (PC) axes account for over half of the shape variation in both lateral and ventral cranial views (PC1_lateral_ = 43.0 %, PC2_lateral_ = 12.8 %; PC1_ventral_ = 50.2 %, PC2_ventral_ = 9.5 %; Fig. 3a–f). The remaining PCs each explained less than 7 % of variation. These results, and their relation to the digging modes known for *Thomomys* taxa, are summarized in Fig. 3g.

The lateral cranial PC1 divides the two *Thomomys* subgenera: the larger-bodied subgenus *T. Megascapheus* taxa (reds and yellows) have more procumbent incisors (Fig. 3a, c), and the smaller subgenus *T. Thomomys* taxa (blues and greens) have less procumbent incisors (Fig. 3a, c). The distribution of taxa along ventral cranial PC1 is similar to their distribution along the lateral cranial PC1 with larger body size corresponding to deeper, more robust skulls (Fig. 3d, f) and smaller body size corresponding to more gracile skulls (Fig. 3d, f). On the PCA graphs of lateral and ventral cranial shape, subspecies of *T. (Thomomys) talpoides* (blues) and *T. (Megascapheus) bottae canus* (light yellow; note that this taxon is an unfortunately named member of the *T. M.* Townsendii clade) occupy the intermediate morphospace between the two subgenera (Fig. 3c, f). In summary, the lateral and ventral PC1 axes appear to reflect allometrically increased incisor procumbency and cranial robusticity, respectively.

The lateral cranial PC2 distinguishes between the taxa of the two subgenus *Megascapheus* clades, *T. M.* Bottae (reds) *versus T. M.* Townsendii (yellows) (Fig. 3c, f). This divergence is based on *T. M.* Bottae taxa (reds) having distally displaced infraorbital foramens relative to *T. M.* Townsendii taxa (yellows) in addition to increased procumbency (Fig. 3b). Furthermore, the lateral PC2 almost completely separates the two smallest *T. (T.) talpoides* subspecies (blues) from the rest of subgenus *T. Thomomys* clade (greens). Similar to the *T. Megascapheus* divergence, this divergence within the small-bodied subgenus *T. Thomomys* is associated with increased procumbency and a distally displaced infraorbital foramen (Fig. 3b, c). The distal movement of the infraorbital foramen in both large and small taxa suggests that allometry-independent cranial rearrangement may underlie increased procumbency along this axis.

The ventral cranial PC2 also distinguishes between the taxa of the two subgenus *T. Megascapheus* clades (reds *versus* yellows; Fig. 3e, f). This divergence in the ventral view is based on the greater size of ventral muscle attachment sites in *T. M.* Bottae taxa (reds) *versus* a dorsal-shift in the orientation of the foramen magnum in *T. M.* Townsendii taxa (yellows) (Fig. 3e). The largest taxa in the genus, *T. (M.) townsendii,* has the most dorsally shifted foramen magnum (Fig. 3e). Unlike the lateral PC2 results, however, procumbent *T. Thomomys* taxa (blues) do not diverge from non-procumbent sister taxa (greens) in either foramen magnum location or ventral muscle attachment sites (Fig. 3f). In summary, the distribution of taxa along lateral cranial PC2 do not always correspond to their distribution along ventral cranial PC2.

### Variation in cranial allometry

**Fig. 4:**
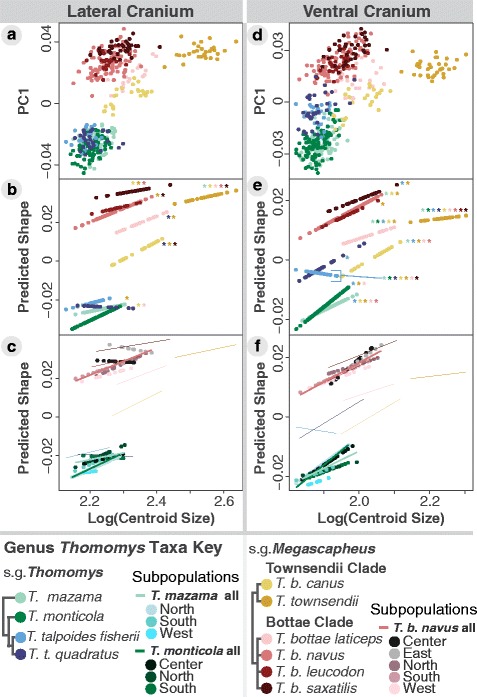
Taxa-specific cranial allometric slopes as a function of centroid size. Centroid size is a proxy for body size. **a** and **d** show PC1 *versus* centroid size for lateral and ventral cranial views, respectively. The remaining graphs **b**, **c**, **e**, **f** use the first principal component of the predicted shape scores from the multivariate regression against log-transformed centroid size (after [53]). The bottom graphs **c** and **f** show the variation in allometric slopes within regional subpopulations sufficiently sampled to be comparable to the species and subspecies slopes from **b** and **e**, respectively, shown as lines to reduce visual clutter

The MANCOVAs confirmed a significant association between size and shape in both cranial views (P_lateral_ = 0.001; P_ventral_ = 0.001). Size explained about 20 % of the shape variation while phylogenetic affiliation (“taxa”; Fig. 1) explained about 38 % (Table 1). Furthermore, the MANCOVAs confirmed significant interaction terms between size and taxa for both cranial views (P_lateral_ = 0.001; P_ventral_ = 0.001; Table 1; Fig. 4b, e), indicating that allometric slopes differ between taxa. Subsequent pairwise slope tests (Fig. 4b, e) showed more instances of significant taxa-specific allometric slope divergences in the ventral view than in the lateral view. These results suggest that the allometric relationship between size and incisor procumbency is less labile than that between size and skull robustness (indicated by fewer significant values in Table 2 *versus* Table 3, Fig. 4b *versus* e). In other words, the allometric patterning of ventral view muscle attachment sites and of foramen magnum location appears to have greater variation than the allometric patterning of incisor procumbency. This is consistent with the results from the cranial shape PCAs.

**Table 1:** Examining static allometry: MANCOVAs of cranial and humeral shape by size and taxa (Y ~ size*taxa)

	Df	SS	MS	R^2^	F	P
Lateral Cranial Shape						
log (size)	1	0.14527	0.145267	0.20361	203.2500	**0.001**
taxa	9	0.27228	0.030253	0.38163	42.3282	**0.001**
log (size):taxa	9	0.01002	0.001113	0.01405	1.5579	**0.001**
residuals	400	0.28589	0.000715			
total	419	0.71345				
Ventral Cranial Shape						
log (size)	1	0.16758	0.167576	0.20197	222.7192	**0.001**
taxa	9	0.32645	0.036273	0.39346	48.2088	**0.001**
log (size):taxa	9	0.01063	0.001182	0.01282	1.5703	**0.001**
residuals	432	0.32504	0.000752			
total	451	0.82970				
Anterior Humeral Shape						
log (size)	1	0.004505	0.0045052	0.07927	9.2624	**0.001**
taxa	8	0.021335	0.0026669	0.37536	5.4828	**0.001**
log (size):taxa	8	0.004246	0.0005307	0.07470	1.0911	0.092
residuals	55	0.026752	0.0004864			
total	72	0.056838				

The effect of centroid size (a proxy for body size) on cranial and humeral shapes within the 10 distinct genus *Thomomys* taxa as evaluated by MANCOVA (details in methods). Degrees of freedom (Df) for each sums of squares (SS) of each term, model residuals, and the total are presented, along with the coefficient of determination (R^2^), and the F ratio and associated *P* value. Statistical significance of the models was evaluated by permutation using 1000 iterations. Bold indicates *p*-values less than 0.05

**Table 2:** Post-hoc test for homogeneity of slopes in the lateral cranial dataset

	*maz*	*mon*	*fis*	*qua*	*can*	*tow*	*lat*	*nav*	*leu*	*sax*
*maz*	–	0.50	0.70	*0.10*	0.40	**0.05**	0.35	0.30	0.30	*0.10*
*mon*	47.0	–	0.70	0.30	0.20	0.20	0.35	0.30	0.55	0.70
*fis*	62.6	59.7	–	0.15	0.30	0.15	0.65	0.35	0.80	0.55
*qua*	60.0	57.1	83.3	–	**0.05**	*0.10*	**0.05**	*0.10*	0.15	*0.10*
*can*	49.5	57.0	67.7	76.3	–	**0.05**	0.30	0.30	0.15	**0.05**
*tow*	65.4	56.5	78.9	72.7	60.5	–	**0.05**	**0.05**	0.15	**0.05**
*lat*	50.3	59.1	63.1	76.0	58.9	77.1	–	0.30	0.65	0.15
*nav*	45.3	46.6	66.6	63.6	45.4	66.5	47.4	–	0.35	**0.05**
*leu*	64.5	60.3	64.3	76.4	68.9	67.8	58.0	61.9	–	0.20
*sax*	61.6	44.3	67.5	69.0	63.2	66.3	64.5	55.4	69.2	–

Post-hoc test table shows the pairwise results testing for homogeneity of slopes (common allometric trajectories). Significance is reported as *p*-values in the upper triangle and angles (degrees) are reported in the lower triangle. Statistical significance rejecting the null hypothesis of a common slope was evaluated by permutation using 1000 iterations. Bold indicates *p*-values less than 0.05. Italics indicate *p*-values between 0.05 and 0.1, suggesting trends for divergence. Taxa are listed in the same order as given by Fig. 1; abbreviations are as follows: *maz = T. (T.) mazama, mon = T. (T.) monticola, fis = T. (T.) talpoides fisherii, qua = T. (T.) talpoides quadratus, can = T. (M.) bottae canus, tow = T. (M.) townsendii, lat = T. (M.) bottae laticeps, nav = T. (M.) bottae navus, leu = T. (M.) bottae leucodon, sax = T. (M.) bottae saxatalis*

**Table 3:** Post-hoc test for ventral cranial allometric slope divergence

	*maz*	*mon*	*fis*	*qua*	*can*	*tow*	*lat*	*nav*	*leu*	*sax*
*maz*	–	*0.09*	**0.01**	0.36	**0.03**	**0.00**	**0.00**	**0.03**	0.65	0.19
*mon*	43.8	–	**0.03**	0.70	0.73	**0.00**	**0.03**	0.17	0.55	0.23
*fis*	82.7	77.5	–	**0.01**	**0.01**	**0.00**	**0.03**	*0.07*	0.18	**0.02**
*qua*	42.2	37.3	86.6	–	0.34	**0.00**	*0.07*	0.09	0.40	0.19
*can*	46.2	32.0	86.2	42.3	–	**0.02**	**0.02**	**0.01**	0.32	0.11
*tow*	78.7	62.3	91.3	69.7	53.1	–	**0.00**	**0.00**	**0.01**	**0.00**
*lat*	56.9	58.6	78.0	58.9	59.3	84.2	–	*0.08*	0.76	0.17
*nav*	35.7	36.1	68.2	46.0	45.2	79.1	47.6	–	0.91	*0.08*
*leu*	41.4	45.9	69.9	52.7	49.4	76.8	44.1	32.8	–	0.83
*sax*	41.5	42.6	79.0	48.7	44.8	72.6	49.7	40.7	39.5	–

Post-hoc test table shows the pairwise results testing for homogeneity of slopes (common allometric trajectories). Significance is reported as *p*-values in the upper triangle and angles (degrees) are reported in the lower triangle. Statistical significance rejecting the null hypothesis of a common slope was evaluated by permutation using 1000 iterations. Bold indicates *p*-values less than 0.05. Italics indicate *p*-values between 0.05 and 0.1, suggesting trends for divergence. Taxa abbreviations as in Table 2

The allometric plots visualizing the variation detected in the MANCOVAs reveal substantial differences in allometric slopes and in intercepts between taxa (Fig. 4). The significant slope differences preclude tests for intercept differences; however, both lateral and ventral view plots show that many taxa separate along the y-axis such that intersections would not occur in the biologically possible morphospace (Fig. 4b, e). For example, the slopes of *T. (M.) b. navus* (red) and *T. (M.) b. canus* (light yellow) would intersect well outside the size range of this rodent genus (Fig. 4b, e).

**Fig. 5:**
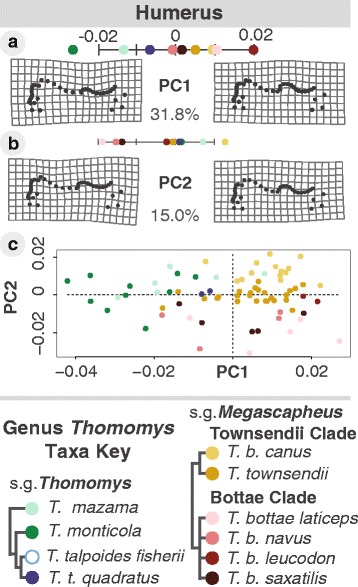
Humeral shape principal component analysis. For all humeral PCs, positive scores correspond with more derived tooth-digging shapes **a**–**c**. Anterior humeral PC1 shows increased deltoid process size **a**. PC2 shows increased medial epicondyle size **b**

Upward shifts in y-intercepts appear to identify the tooth-digging taxa compared to claw-digging relatives (Fig. 3g, Fig. 4b, e; Fig. 5): tooth-digging *T. M.* Townsendii clade taxon, *T. (M.) townsendii* (dark yellow) *versus* claw-digging sister taxon, *T. (M.) b. canus* (light yellow); tooth-digging *T. M.* Bottae clade taxa, *T. (M.) bottae navus*, *T. (M.) b. leucodon*, and *T. (M.) b. saxatalis* (reds) *versus* claw-digging sister taxon, *T. (M.) b. laticeps* (pink); and finally the two tooth-digging subgenus *T. Thomomys* subspecies of *T. (T.) talpoides* (blues) *versus* claw-digging sister taxa, *T. (T.) mazama* and *monticola* (greens) (Fig. 4e)*.* Subgenus *T. Thomomys* taxon, *T. (T.) talpoides quadratus* (dark blue) stands out by displaying more robust crania than much larger individuals of subgenus *T. Mesgascapheus* Townsendii taxon, *T. (M.) b. canus* (yellow) (indicated by parallel slopes in Fig. 4d, e). The lateral cranial view shows the same pattern of y-intercept upward shifts for tooth-digging in *T. Megascapheus* taxa but not in the subgenus *T. Thomomys* taxa (indicated by overlapping blues and greens in Fig. 4b)*.*

Taxa within a clade rarely exhibit significantly different allometric slopes, however, three notable exceptions occur. First, a significant difference between the two *T. Megascapheus* Townsendii taxa appears to reflect the nearly flat allometric slope of *T. (M.) townsendii* (dark yellow) in contrast to the steeper slope of sister taxon *T. (M.) b. canus* (light yellow), seen in both views (Tables 2 and 3; Fig. 4b, e). In support of this, the allometric slope of *T. (M.) townsendii* significantly differs from all other taxa in the ventral view (Table 3, Fig. 4e). A similar effect appears to occur within *T. Megascapheus* Bottae taxa in the significantly different lateral cranial allometric slopes of *T. (M.) b. navus* (light red) and *T. (M.) b. saxatilis* (dark red); these are also the two most procumbent taxa in the genus (Fig. 4b).

The third instance of within-clade significant allometric slope divergence involves the two subspecies of *T. (T.) talpoides* (Table 3; Fig. 4e)*.* In the lateral cranial view, procumbency appears to have less relation to centroid size for *T. (T.) talpoides quadratus* (dark blue) (indicated by significant differences with larger taxa in Table 2 and a flat slope in Fig. 4b), while the ventral cranial view, this taxon shows similar allometric patterns of skull robustness to other *Thomomys* taxa (non-significant values in Table 3; parallel slope to other taxa in Fig. 4e). By contrast, the ventral crania of *T. (T.) talpoides fisherii* (light blue) appear less robust with increasing centroid size (significant values in Table 2; negative slope in Fig. 4e), while its allometric relationship with procumbency is similar to that of the other clades (non-significant values in Table 3; parallel slope in Fig. 4b).

Splitting the dataset into regional subpopulations (Fig. 4c, f) reveals substantial diversity in allometric slopes which clearly contribute to differences in taxa-specific allometric slopes. The sample sizes for these subpopulations, however, are possibly too small for a confident identification of significantly different allometric slopes (Additional file 3: Table S3).

### Variation in cranial shape in relation to soil

**Fig. 6:**
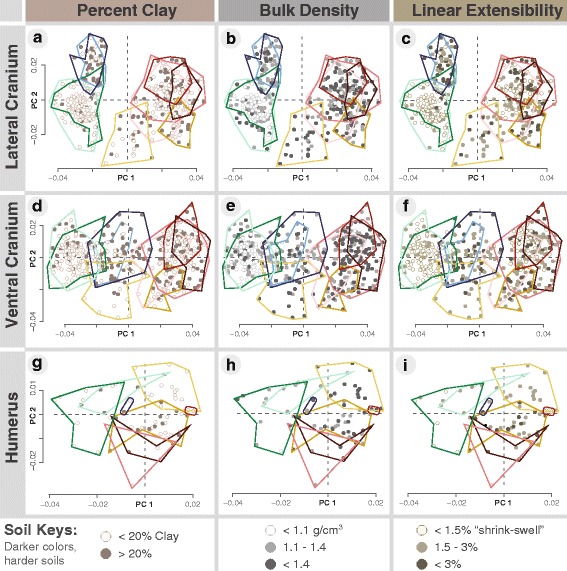
The relationship between cranial and humeral shape with soil type. For all cranial and humeral PCs, positive scores correspond with shapes derived for digging in harder soils; convex hulls are colored according to taxonomy (Fig. 1) **a**–**i**. Points are colored according to soft, medium, and hard for each soil condition: percent clay **a**, **d**, **g**, bulk density **b**, **e**, **h** and linear extensibility **c**, **f**, **i** with bin categories after Marcy et al. 2013 [27]

The PCAs of cranial shape colored by soil type demonstrate that the presumed ancestral taxa, *T. (T.) mazama* and *T. (T.) monticola* (greens) occupy the softest soils in the region (Fig. 6a–f). Their sister taxa, however, (the two subspecies of *T. (T.) talpoides* [blues]) appear to inhabit soils of hardness comparable to the larger subgenus *T. Megascapheus* gophers (reds and yellows) (Fig. 6a–f). This is particularly visible in the lateral cranial PCAs (Fig. 6a–c). The least procumbent *T. Megascapheus* taxon, *T. (M.) b. canus* (light yellow), however, appears to inhabit soils of hardness similar to its more procumbent and robust sister taxon, *T. (M.) townsendii* (dark yellow, Fig. 6a–f).

### Variation in humeral shape and its relation to soil

From the humeral views (n_anterior_ = 73), only the first two anterior PC axes explained a meaningful amount of shape variation (PC1_anterior_ = 31.8 %, PC2_anterior_ = 15.0 %; Fig. 5a–d). The results from the lateral humeral view did not provide conclusive information beyond the anterior humeral view (Additional file 7: Supplementary results: lateral humeral view).

PC1 of anterior humeral shape captured deltoid process size, an important muscle attachment site, relative to the lateral epicondyle (Fig. 5a). The anterior humeral PC2 captured the distance of the deltoid process from the humeral head, the size of the medial epicondyle relative to the articular surface, and the orientation of greater tuberosity (Fig. 5b). All of these shape changes associated with more positive values of PC2 increase the mechanical advantage for digging. Along PC1, the larger subgenus *T. Megascapheus* taxa (reds and yellows) tend to have more robust humeri with larger deltoid processes as compared to the smaller subgenus *T. Thomomys* (blues and greens) (Fig. 5a, c). The claw-digging *T. Megascapheus* Townsendii taxa, *T. (M.) b. canus* scores highly along both axes, meaning all of the claw-digging muscle attachment sites highlighted above have increased in relative size.

In comparison to cranial shape, the MANCOVA for humeral shape and size showed that a smaller proportion of shape (less than 8 %, compared to over 20 % in both cranial views) is explained by size (Table 1). Tests for differences in static allometry between taxa were all non-significant (Table 1). Interestingly, while the tooth-digging *T. M.* Bottae taxa have slightly larger skulls than subgenus *Thomomys* taxa (Fig. 4), these two clades have humeri of overlapping centroid sizes (Additional file 8: Figure S2).

**Fig. 7:**
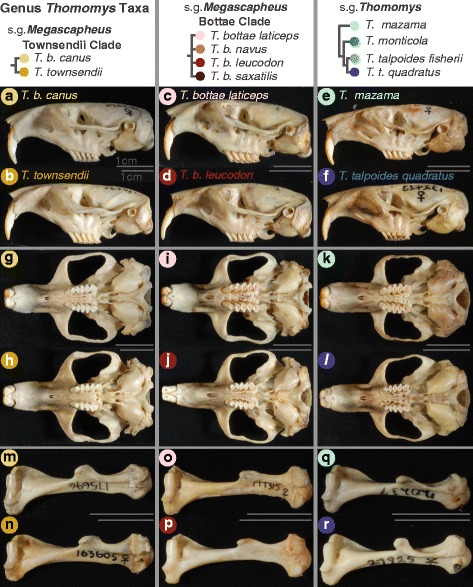
Comparisons of claw-digging to tooth-digging taxa in each major clade. Representatives of claw-digging and of tooth-digging, respectively taxa in each major taxonomic clade. Lines on the right of each image give 1 cm scale for each specimen. Lateral cranial views of *T. M.* Townsendii clade gophers, *T. (M.) b. canus*, a claw-digging gopher found in heavy but friable soils (see also Table 4) **a** *versus* its sister taxa *T. (M.) townsendii*, a tooth-digging species also found in heavy but friable soils **b**. Lateral cranial views of *T. M.* Bottae clade gophers, *T. (M.) bottae laticeps*, a claw-digging taxa found in friable clay soils **c** *versus* its tooth-digging sister subspecies, *T. (M.) bottae leucodon*, one of the most procumbent taxa in the genus **d**. Lateral cranial view of subgenus *Thomomys* gophers, *T. (T.) mazama*, representing the most ancestral claw-digging condition in very soft soils **e** *versus* its sister taxon, *T (T.) talpoides quadratus*, which tooth-digs in some of the hardest soils in the region **f**. Ventral cranial views for the same taxa **g**–**l**. Anterior humeri views for the same taxa **m**–**r**

The first quadrants of the PCAs of humeral shape colored by soil type show that the most derived humeral shape belongs to the non-procumbent taxon *T. (M.) b. canus* (light yellow), which nonetheless inhabits hard soils (Fig. 6h, i). The least procumbent *T. M.* Bottae clade taxon, *T. (M.) b. laticeps* is found in similar soils as *T. (M.) b. canus* and the two taxa converge on a larger deltoid crest (Figs. 5, 6 and 7). The second quadrant captures a humeral shape associated with non-procumbent taxa inhabiting soft soils (Fig. 6g–i). The two bottom quadrants appear to capture a humeral shape of taxa with the ability to dig with their teeth (Fig. 6g–i). The smallest species in the genus, *T. (T.) talpoides* and the largest species in the genus, *T. (M.) townsendii* are intermediate between the humeral shape of species known to specialize in claw-digging and those that have adaptations for tooth-digging (Fig. 6; Table 4).

**Table 4:** Summary of interpretation of adaptation strategies given shape, soil, and body size

Taxa	Soil type occupied	Digging strategy used	Evolutionary comments
*T (T.) mazama*	Soft sandy soil - low clay, bulk density, and linear extensibility; high sand make this relatively easy to dig in	Ancestral claw-digging	Likely illustrates the primitive ancestral condition except in lateral humeral shape.
*T (T.) monticola*	Soft sandy soil - lowest clay, bulk density, and linear extensibility; highest sand in the genus	Ancestral claw-digging	Arguably the easiest soil in the region, likely preserves the primitive ancestral condition for the genus.
*T (T.) talpoides fisherii*	Friable clay soil - medium clay but high sand and low bulk density suggests it is not very compacted	Derived tooth-digging despite body size	While still appears to tooth-dig, like sister subspecies below, may be shifting back towards claw-digging in sandier soils
*T (T.) talpoides quadratus*	Hard clayey soil - high clay and very low sand, low bulk density suggests it is not very compacted	Derived tooth-digging despite body size	A combination of allometric and non-allometric cranial rearrangement appears to produce derived tooth-digging shape
*T. (M.) b. canus (Townsendii clade)*	Heavy sandy soil - low clay and high sand suggests the latter drives high bulk density	Claw digging despite body size	Intermediate soil appears to have selected for more for claw-digging adaptations
*T. (M.) townsendii*	Heavy sandy soil - more clay than sister species above but still high sand	Tooth-digging via size-increase alone	In contrast to sister species above, intermediate soil appears to have selected more for tooth-digging adaptations
*T. (M.) bottae laticeps*	Friable clay soil - medium clay but high sand and low bulk density make it easier to manipulate	Claw-digging despite body size	Diverging from the rest of its clade, intermediate soil appears to have selected for claw-digging adaptations
*T. (M.) bottae navus*	Friable clay soil - medium clay and medium bulk density but high sand make it easier to manipulate	Derived tooth-digging	In contrast to sister subspecies above, intermediate soil appears to have selected for tooth-digging adaptations
*T. (M.) bottae leucodon*	High clay soil - highest clay and linear extensibility with low sand and low bulk density	Derived tooth-digging	Arguably the hardest soils in the region appear to have selected for both tooth- and claw-digging adaptations
*T. (M.) bottae saxatilis*	High clay soil - medium clay and linear extensibility with low sand and high bulk density	Derived tooth-digging	In slight contrast to sister subspecies, hard soils appear to have selected for a slightly more procumbent tooth-digging shape & less emphasis on claw-digging

Values for soil conditions that impact digging. Percent clay is the part of soil texture that confers plasticity, and in high amounts, makes soil difficult to manipulate. Percent sand is the heaviest part of soil texture, and in high amounts makes soil heavy but easy to break apart. Bulk density is an indicator of soil compaction calculated by the dry weight of soil divided by its volume—it can have high values due to compacted clay, or to a high percent of sand, the heaviest component of soil texture. Linear extensibility, a property of certain kinds of clay, quantifies the shrink-swell capacity of soil. This property causes soils to harden when dry, warm climatic conditions reduce the effective moisture in the soil

## Discussion

Our results reveal that different combinations of intrinsic shape-change processes appear to resolve conflicts of form and function presented by the rodent skeleton in the context of fossorial selection pressures. In genus *Thomomys*, body size change seems to mediate allometric shape changes which are likely adaptive in harder soils, with several exceptions. At the species and subspecies level, cranial and humeral shapes appear to exhibit finely distinguished adaptations to local soil conditions (Table 4). Furthermore, we identify several taxa pairs that inhabit soils presenting similar digging challenges yet exhibit diverging morphologies. This suggests a trade-off between procumbent tooth-digging shapes and body size that each of the three main clades balances differently. Together, these processes seem to generate the remarkable morphological diversity of this genus in which each even sister subspecies can diverge substantially, and distant relatives may converge on similar morphologies.

### Procumbency evolves through changes in allometry and can interact with wholesale cranial re-arrangements

The genus *Thomomys* contains three clades, each appearing to evolve the procumbent condition required for prolonged tooth-digging in hard soils through different variations on two intrinsic processes: body size allometry resulting in a larger incisor arc and cranial rearrangements resulting in a more posterior incisor position (Fig. 7). Illustrating the first variant, procumbency in the largest member of genus *Thomomys*, *T. (M.) townsendii*, appears to have evolved through marked body size increase with minimal or no cranial rearrangement (Figs. 5 and 7). Interestingly, static allometric slopes vary widely across taxa; in particular, *T. (M.) townsendii* has the shallowest slope for its subgenus (Fig. 4). This might be explained if this species is at the limit of what allometry-related procumbency can produce alone.

Illustrating the second proposed variant of procumbency evolution, taxa in the sister clade to *T. M.* Townsendii, *T. M.* Bottae, appear to derive their extreme incisor procumbency through body size increase as well as incisor root posterior positioning. The combination of both static allometry and cranial rearrangements results in the greatest degree of procumbency within the genus and in fact the entire family of Geomyidae [23, 55]. It is possible that having a more procumbent yet smaller body size than *T. (M.) townsendii* may allow *T. M.* Bottae taxa to dig in the hardest soils of the genus (Figs. 3, 6 and 7; Table 4). The phylogeny suggests that cranial rearrangements evolved quickly, in less than 1.93 Ma, which is the divergence date known for the split preceding the split between the *T. M.* Bottae and the *T. M.* Townsendii clades [20]. Similar to the pattern seen in *T. (M.) townsendii*, the most procumbent *T. M.* Bottae clade taxa, *T. (M.) b. saxatilis* also has a significantly more shallow allometric slope (Fig. 5), which suggests their shared morphology imposes an upper limit on size-related procumbency.

The third proposed variant of procumbency evolution, solely produced through cranial rearrangement, appears to underlie a previously unreported case of convergent evolution between subgenera, *T. Thomomys* and *T. Megascapheus.* Our results suggest that the smallest species in the genus, *T. (T.) talpoides* tooth-digs in soils of similar hardness as most *T. M.* Bottae clade taxa (Table 4; Figs. 6 and 7). Unlike *T. M.* Bottae clade taxa and *T. (M.) townsendii*, however, *T. (T.) talpoides* only displays a posteriorly shifted incisor root, while their strikingly shallow allometric slopes do not support an allometric process underlying their procumbency (Figs. 3, 5 and 7). As a result, *T. (T.) talpoides*’s cranial shape more closely resembles that of taxa from the much larger, hard-soil-digging *T. M.* Bottae clade than their closest relative, *T. (T.) monticola* (Fig. 7; Table 4). Again, the phylogeny suggests this change evolved relatively quickly: in less than 2.67 Ma, the divergence date for the split preceding the *T. (T.) monticola* and *T. (T.) talpoides* split (Fig. 1) [20]. Indeed, PC2 shows that the incisor position of *T. (T.) talpoides* is far more posterior and thus more derived than the *T. M.* Bottae clade (Fig. 3), possibly explaining its divergence from allometric slopes seen in most other genus *Thomomys* taxa, particularly in the ventral view (Fig. 4).

### Allometric trajectories vary substantially among finely distinguished taxonomic levels

As the divergent allometric patterning within *T. (T.) talpoides* illustrates, a generalization from this study is that static allometric slopes of cranial shape can vary widely within one genus and even between subspecies. We found that the allometric slopes of regional subpopulations even within a single subspecies, like *T. (M.) bottae navus,* can vary dramatically. Indeed, as Fig. 4c, f show, populations of this one subspecies display all three kinds of allometric slopes seen across the genus (general pattern, steeper slopes, and shallower slopes, including “negative” slopes). It is unclear whether these allometric slope variations represent rapid adaptation events of shape due to different soil conditions *in situ*, to soils they no longer occupy or that may not exist presently, or whether they are due to founder effects and genetic drift. The striking variation within this single subspecies, however, reveals the considerable diversity of shape provided through allometric processes readily available to natural selection.

The variation among intraspecific allometric slopes emphasizes the divergence between high-resolution patterns such as found here and larger-scale patterns of phylogenetically more-inclusive comparative studies. For example, an averaged subgenus *T. Megascapheus* slope would mask the *T. M.* Bottae clade’s increased y-intercept reflecting cranial rearrangement as well as the shallower slopes that appear to correspond with limits on absolute procumbency in *T. (M.) b. saxatalis* and *T. (M.) townsendii*. Studies that quantify morphology at higher taxonomic levels should thus acknowledge the possible loss of informative and potentially divergent allometric patterns visible at finer taxonomic resolution.

### Post-cranial adaptation for claw-digging may reduce selection on the skull and represent an additional mechanism of fossorial adaptation

Our analyses of humeral shape suggest that claw-digging adaptations present less of a trade-off between body size and digging ability compared to cranial adaptations of procumbency [56]. Indeed, while the tooth-digging *T. M.* Bottae taxa have slightly larger skulls than subgenus *Thomomys* taxa, these two clades have humeri of overlapping centroid sizes. Perhaps the bulky muscles associated with claw-digging and the larger heads associated with tooth-digging both contribute to burrow diameter [26] but limb size may be less coupled to burrow diameter than is skull size.

Some humeral shape variations correspond with soil type, suggesting that the humerus is also under differential soil-related natural selection. Indeed, in soils of intermediate hardness, more derived humeral shapes appear to reduce selection for more derived skull shapes. For example, the two sister taxa within the *T. M.* Townsendii clade, *T. (M.) b. canus* and *T. (M.) townsendii*, occupy similarly hard soils but show opposing shape trends in the humeri and in the crania: the former, smaller taxon scores highly on humeral PC axes but low on cranial PC axes, while these trends are reversed in the latter, larger taxon. The derived humeral shape also appears to explain how non-procumbent *T. (M.) b. canus* can inhabit soils of hardness similar to the more procumbent and robust taxa in subgenus *T. Megascapheus* (e.g. compare Fig. 6b and h). Similarly, the least procumbent *T. M.* Bottae clade taxon, *T. (M.) bottae laticeps* also scores highly on anterior humeral PC1 (meaning it has a larger deltoid process). Thus *T. (M.) b. laticeps* and *T. (M.) b. canus* appear to demonstrate an instance of convergence in deltoid process shape between the two clades of subgenus *T. Megascapheus*. On the other hand, *T. (M.) bottae leucodon*—which occupies the hardest soils in the region—has both a highly procumbent cranial shpae and the highest average humeral PC1 shape (i.e. the largest deltoid process relative to centroid size) within the genus. These results suggest that both cranial and humeral adaptation can concurrently evolve.

The evolution of humeral shape appears to occur in diverse ways across the genus *Thomomys*, with little evidence for any particular sequence of adaptation to fossoriality. This contradicts a previous suggestion of a two-step adaptive pathway for claw-digging in rodents based on humeral shape in the fossorial rodent genus *Ctenomys* (see [42])*.* According to this scenario, an enlarged deltoid and epicondylar crests would first co-occur with fossorial habits and then an increased articular surface would co-occur with harder soils [42]. However, our findings suggest that the size of the deltoid is more indicative of claw-digging in hard soils (Fig. 5a; Fig. 7m–r; Table 4), while the largest articular surface belongs to *T. (T.) mazama* which digs in softest soils (Fig. 5g; Fig. 7q). In *Ctenomys*, the ancestral positioning of the incisor root is lateral to the cheek teeth, which could possibly decrease the constraints on evolutionary rearrangements of the incisor root. It is plausible that this could result in a lower selection pressure on humeral fossorial adaptation in *Ctenomys*, resulting in different evolutionary patterning in this genus compared to *Thomomys*. A larger sample of *T. (T.) talpoides* humeri (*n* = 2) in our analysis could provide an intriguing comparison patterns of humeral *versus* cranial evolution in hard soil. Regardless, our results suggest that chance and phylogenetic history have greater roles in fossorial adaptation than any intrinsic rodent pathway to claw-digging.

### Static allometry suggest ontogenetic allometry studies could reveal different heterochronic mechanisms underlying convergent shapes in genus *thomomys*

Our study is limited to adult specimens, thus the static allometry we discuss here cannot adequately test for ontogenetic mechanisms underlying the evolution of diverse cranial shapes in *Thomomys*. Past literature on this genus [33] and other rodents [40, 41] have concluded from the study of static allometry that peramorphosis—i.e. exaggeration of adult shapes through sustained growth—underlies the increased incisor procumbency via body size increase. These scenarios have assumed that procumbency evolves through a single peramorphic developmental mechanism, which would result in a single allometric slope for all taxa. Our results show, however, that taxa have different static allometric trajectories—even among closely related subspecies—and therefore suggest a more complex scenario. In particular, the allometric slopes of the two subspecies of *T. (T.) talpoides* diverge noticeably from the presumed “peramorphic” pattern seen in the rest of the genus. It is possible that the shallower slopes we detect via static allometry might reflect a paedomorphic process—i.e. the retention of juvenile shapes as adults. This potential contrast to the peramorphic process most likely underlying allometric procumbency in the other pocket gophers presents a promising investigation into the evolutionary mechanisms underlying convergent and parallel evolution (e.g. [57, 58])*.*

## Conclusions

We conducted geometric morphometric analyses on a diverse pocket gopher genus from a small geographic region with fine-grained taxonomic distinctions. Our results revealed that both allometry and differential evolution of functional cranial *versus* humeral shapes appear to have generated substantial differences in the digging apparatus essential for life underground. Because the fossorial niche exerts a high-energy-cost selection pressure [18], functional trade-offs, such as between tooth-digging and tunnel size, as well as biological constraints, particularly of body size, appear to channel the skull into a limited morphospace. The resulting relationship between shape and body size, however, appears more complex than the previously suggested uniform allometric mechanisms proposed earlier [28]. The results also reveal substantial adaptive flexibility within the genus, for example in our newly reported instance of convergent evolution in the smallest-bodied gopher *T. (T.) talpoides*. In this species, allometric constraints appear to be circumvented through evolution of the most derived cranial rearrangement for tooth-digging seen in the genus.

In a recent meta-analysis of convergent evolution, Ord & Summers (2015) [2] report that repeated evolution of similar morphological traits is much more common in closely related taxa [2]. Their assumed mechanism is that shared genomic and/or developmental pathways produce similar morphological changes in similar ways. Our results, however, suggest that the incisor procumbency in *T. (T.) talpoides* evolved in a different way than the procumbency in *T. Megascapheus* gophers. Therefore, this genus of pocket gophers seems to provide cases of parallel evolution (allometry between subgenus *T. Megascapheus* taxa) and a case of convergent evolution in which the similar trait is produced by different combinations of processes (allometry and/or cranial rearrangement). Our suggestion provides an intriguing addition to the mechanisms for repeated evolution proposed by Ord & Summers (2015) [2], particularly in species with pronounced developmental constraints living under a strong selection pressure. The developmental mechanisms underlying the allometric and the cranial rearrangement processes producing incisor procumbency, which have been frequently invoked in the past literature (e.g. [28]), will require further analyses with ontogenetic series.

Genus-level adaptive lability is apparent in the fact that each of the three distinct clades harbor at least one taxon diverging from its sister taxa along the spectrum of claw-digging to tooth-digging. Phylogenetic analyses suggest that these adaptations evolved in short evolutionary timescales of around 2 Ma [20]. The diversity of digging adaptions across the phylogeny of genus *Thomomys* suggests that taxa from each of the three clades could opportunistically occupy and adapt to any regional soil type if a neighboring gopher species went locally extinct. This “niche conservatism” appears to have acted in changing climate conditions during the Pleistocene-Holocene transition, which coincided with a species turn-over event in our study area [59]. In this case, a tooth-digging species replaced a claw-digging species [59]; the former appears to have had an advantage in the drier climate [27]. Because species turn-over events like this still maintain the genus-wide range, we propose that within-clade diversity in functional shapes, as detected in this study, underlies the evidence for genus-level niche conservatism inferred from stable mammal genera range sizes [60].

## Additional files

Additional file 1: Table S1. Specimen numbers for analyses by view and phylogenetic affiliation. (XLSX 482 kb)
Additional file 2: Table S3. MVZ specimen catalog numbers and views represented. (XLSX 495 kb)
Additional file 3: Table S4. Regional sub-population sample sizes by subspecies and cranial view. (XLSX 482 kb)
Additional file 4: Supplementary methods. (DOCX 79 kb)
Additional file 5: Figure S1. Landmark and semilandmark key. Red circles indicate location of landmarks, blue lines indicate sliding paths for semilandmarks on lateral cranial view **a**, ventral cranial view **b**, anterior humeral view **c**, and lateral humeral view **d**. Definitions in Additional file 6: Table S4. (PNG 2510 kb)
Additional file 6: Table S3. Definitions of landmarks. (XLSX 492 kb)
Additional file 7: Lateral humeral shape results. (DOCX 75 kb)
Additional file 8: Figure S2. Multivariate regression plots. The regression score (after [55]) of lateral **a** and ventral **b** cranial shape *versus* log centroid size show the allometric variation across all taxa. These data underlie the predicted shape allometric slopes in Fig. 4b and e, respectively. Regression score plot for humeral data **c** demonstrates that the *T. M.* Bottae clade taxa and the subgenus *Thomomys* taxa have similar centroid sizes. (PNG 274 kb)

## Abbreviations

*can*: *T. (M.) bottae canus*
*fis*: *T. (T.) talpoides fisherii*
*lat*: *T. (M.) bottae laticeps*
*leu*: *T. (M.) bottae leucodon*
LM: Landmark
Ma: Million years ago
*maz*: *T. (T.) mazama*
*mon*: *T. (T.) monticola*
*nav*: *T. (M.) bottae navus*
PC: Principal Component
PCA: Principal Component Analysis
*qua*: *T. (T.) talpoides quadratus*
*sax*: *T. (M.) bottae saxatalis*
*T. (M.)*: Genus *Thomomys* subgenus *Megascapheus*
*T. (T.)*: Genus *Thomomys* subgenus *Thomomys*
*tow*: *T. (M.) townsendii*

## References

[1] Drake AG, Klingenberg CP (2010). Large-Scale Diversification of Skull Shape in Domestic Dogs: Disparity and Modularity. Am Nat.

[2] Ord TJ, Summers TC (2015). Repeated evolution and the impact of evolutionary history on adaptation. BMC Evol Bio..

[3] Klingenberg CP (1998). Heterochrony and allometry: the analysis of evolutionary change in ontogeny. Biol Rev.

[4] Klingenberg CP (2016). Size, shape, and form: concepts of allometry in geometric morphometrics. Dev Genes Evol..

[5] Wagner GP, Altenberg L (1996). Perspective: Complex adaptations and the evolution of evolvability. Evolution.

[6] Uzum N, Ivanovic A, Gumus C, Avci A, Olgun K (2015). Divergence in size, but not in shape: variation in skull size and shape within *Ommatotriton* newts. Acta Zool.

[7] Franchini P, Colangelo P, Meyer A, Fruciano C (2016). Chromosomal rearrangements, phenotypic variation and modularity: a case study from a contact zone between house mouse Robertsonian races in Central Italy. Ecol Evol.

[8] Weisbecker V, Goswami A, Wroe S, Sanchez-Villagra MR (2008). Ossification heterochrony in the therian postcranial skeleton and the marsupial-placental dichotomy. Evolution.

[9] Ross D, Marcot JD, Betteridge KJ, Nascone-Yoder N, Bailey CS, Sears KE (2013). Constraints on mammalian forelimb development: insights from developmental disparity. Evolution.

[10] Polyakov A, Beharav A, Avivi A, Nevo E (2004). Mammalian microevolution in action: adaptive edaphic genomic divergence in blind subterranean mole-rats. Proc R Soc B.

[11] Carmona FD, Jimenez R, Collinson JM (2008). The molecular basis of defective lens development in the Iberian mole. BMC Biol..

[12] Nevo E (2011). Evolution Under Environmental Stress at Macro- and Microscales. Genome Biol Evol.

[13] Tomasco IH, Lessa EP (2011). The evolution of mitochondrial genomes in subterranean caviomorph rodents: Adaptation against a background of purifying selection. Mol Phylogenet Evol.

[14] Lovy M, Skliba J, Burda H, Chitaukali WN, Sumbera R (2012). Ecological characteristics in habitats of two African mole-rat species with different social systems in an area of sympatry: implications for the mole-rat social evolution. J Zool.

[15] Echeverria AI, Becerra F, Vassallo AI (2014). Postnatal ontogeny of limb proportions and functional indices in the subterranean rodent *Ctenomys talarum* (Rodentia: Ctenomyidae). J Morph.

[16] Stein B. Morphology of Subterranean Rodents. In: Lacey AP, Patton JL, Cameron GN, editors. Life Underground: The Biology of Subterranean Rodents*.* Chicago: University Of Chicago Press; 2000. p. 19–60.

[17] Andersen DC, Macmahon JA (1981). Population dynamics and bioenergtics of a fossorial herbivore, *Thomomys talpoides* (Rodentia, Geomyidae), in a spruce tree sere. Ecol Monogr.

[18] Vleck D. The energy cost of burrowing by the pocket gopher *Thomomys bottae*. Phys Zool. 1979; 52:122–36.

[19] Patton JL. Population structure and the genetics of speciation in pocket gophers, genus *Thomomys*. Acta Zool-Fennica.1985;170:109–114.

[20] Belfiore NM, Liu L, Moritz C (2008). Multilocus phylogenetics of a rapid radiation in the genus *Thomomys* (Rodentia: Geomyidae). Syst Biol.

[21] Patton JL. The evolutionary dynamics of the pocket gopher *Thomomys bottae*, with emphasis on California populations. Berkeley: University of California Press; 1990.

[22] Thaeler C. An analysis of the distribution of pocket gopher species in northeastern California (Genus T*homomys*). Berkeley: University of California Press; 1968.

[23] Lessa EP, Thaeler CS (1989). A reassessment of morphological specializations for digging in pocket gophers. J Mamm.

[24] Buffenstein R. Ecophysiological Responses of Subterranean Rodents to Underground Habitats. In: Lacey AP, Patton JL, Cameron GN, editors. Life Underground: The Biology of Subterranean Rodents*.* Chicago: University Of Chicago Press; 2000. p. 76–81.

[25] Busch C, Antinuchi CD, del Valle JC, Kittlein MJ, Malizia AI, Vassallo AI, et al. Population Ecology of Subterranean Rodents. In: Lacey AP, Patton JL, Cameron GN, editors. Life Underground: The Biology of Subterranean Rodents*.* Chicago: University Of Chicago Press; 2000. p. 183–226.

[26] White CR (2005). The allometry of burrow geometry. J Zool.

[27] Marcy AE, Fendorf S, Patton JL, Hadly EA. Morphological adaptations for digging and climate-impacted soil properties define pocket gopher (*Thomomys* spp.) distributions. PLOS One. 2013; doi:10.1371/journal.pone.0064935. 10.1371/journal.pone.0064935 PMC3663803 23717675

[28] Lessa EP, Patton JL (1989). Structural constraints, recurrent shapes, and allometry in pocket gophers (genus *Thomomys*). Zool J Linn Soc.

[29] Luna F, Antinuchi CD (2006). Cost of foraging in the subterranean rodent *Ctenomys talarum*: effect of soil hardness. Can J Zool.

[30] Romanach SS, Seabloom EW, Reichman OJ (2007). Costs and benefits of pocket gopher foraging: Linking behavior and physiology. Ecology.

[31] Sedláček F, Begall SB, Burda H, Schleich CE (2007). New Data on Metabolic Parameters in Subterranean Rodents. Subterranean Rodents: News from Underground.

[32] Mora M, Olivares AI, Vassallo AI (2003). Size, shape and structural versatility of the skull of the subterranean rodent *Ctenomys* (Rodentia, Caviomorpha): functional and morphological analysis. Zool J Linn Soc.

[33] Landry SO (1957). Factors Affecting the Procumbency of Rodent Upper Incisors. J Mamm.

[34] Anderson PSL, Renaud S, Rayfield EJ. Adaptive plasticity in the mouse mandible. BMC Evol Biol. 2014;14:85. 10.1186/1471-2148-14-85 PMC4002541 24742055

[35] Zumwalt A (2006). The effect of endurance exercise on the morphology of muscle attachment sites. J Exp Biol.

[36] Campione NE, Evans DC (2012). A universal scaling relationship between body mass and proximal limb bone dimensions in quadrupedal terrestrial tetrapods. BMC Biol..

[37] Rabey KN, Green DJ, Taylor AB, Begun DR, Richmond BG, Mcfarlin SC (2015). Locomotor activity influences muscle architecture and bone growth but not muscle attachment site morphology. J Hum Evol.

[38] Verts BC, Carraway LN. *Thomomys talpoides*. Mamm Species. 1999; 618:1–11.

[39] Verts BJ, Carraway LN. *Thomomys townsendii.* Mamm Species 2003; 719 (719):1–6.

[40] Daly JC, Patton JL (1986). Growth, reproduction, and sexual dimorphism in *Thomomys bottae* pocket gophers. J Mammal.

[41] Grieco TM, Rizk OT (2010). Cranial shape varies along an elevation gradient in Gambel’s white-footed mouse (*Peromyscus maniculatus gambelii*) in the Grinnell Resurvey Yosemite Transect. J Morph.

[42] Steiner-Souza F, De Freitas TRO, Cordeiro-Estrela P (2010). Inferring adaptation within shape diversity of the humerus of subterranean rodent *Ctenomys*. Zool J Linn Soc.

[43] Rohlf F. tpsDig, digitize landmarks and outlines. Version 2.10. Stony Brook: Department of Ecology and Evolution, State University of New York, Stony Brook; 2016. http://life.bio.sunysb.edu/morph/

[44] Fernandes FA, Fornel R, Cordeiro-Estrela P, Freitas TRO (2009). Intra- and interspecific skull variation in two sister species of the subterranean rodent genus *Ctenomys* (Rodentia, Ctenomyidae): coupling geometric morphometrics and chromosomal polymorphism. Zool J Linn Soc.

[45] Adams DC, Otarola-Castillo E (2013). *geomorph*: an R package for the collection and analysis of geometric morphometric shape data. Methods Ecol Evol.

[46] Adams D, Collyer ML, Sherratt E. *geomorph*: Software for geometric morphometric analyses. R package version 3.0. 2016. https://CRAN.R-project.org/package=geomorph.

[47] Rohlf FJ, Slice D (1990). Extensions of the Procrustes method for the optimal superimposition of landmarks. Syst Zool.

[48] Gunz P, Mitteroecker P, Bookstein FL. Semilandmarks in three dimensions. In D. E. Slice (Ed.), Modern morphometrics in physical anthropology. New York: Kluwer Academic/Plenum; 2005. pp. 73–98.

[49] R Development Core Team. R: a language and environment for statistical computing. Version 3.2.3. Vienna; 2015. http://www.R-project.org.

[50] Bookstein FL. Morphometric tools for landmark data: geometry and biology. Cambridge: Cambridge University Press; 1991.

[51] Goodall C (1991). Procrustes methods in the statistical analysis of shape. J R Stat Soc B Methodol.

[52] Collyer ML, Sekora DJ, Adams DC (2015). A method for analysis of phenotypic change for phenotypes described by high-dimensional data. Heredity.

[53] Adams DC, Nistri A (2010). Ontogenetic convergence and evolution of foot morphology in European cave salamanders (Family: Plethodontidae). BMC Evol Bio..

[54] Drake AG, Klingenberg CP. The pace of morphological change: historical transformation of skull shape in St Bernard dogs. Proc R Soc B. 2008; doi:10.1098/rspb.2007.1169. 10.1098/rspb.2007.1169 PMC2562403 17956847

[55] Lessa EP, Stein BR (1992). Morphological constraints in the digging apparatus of pocket gophers (Mammalia, Geomyidae). Biol J Linn Soc.

[56] McIntosh AF, Cox PG. The impact of digging on craniodental morphology and integration. J Env Biol. 2006; doi:10.1111/jeb.12962 10.1111/jeb.12962 27521516

[57] Drake AG (2011). Dispelling dog dogma: an investigation of heterochrony in dogs using 3D geometric morphometric analysis of skull shape. Evol Dev.

[58] Strelin MM, Benitez-Vieyra S, Fornoni J, Klingenberg CP, Cocucci AA (2016). Exploring the ontogenetic scaling hypothesis during the diversification of pollination syndromes in *Caiophora* (Loasaceae, subfam. Loasoideae). Ann Bot.

[59] Blois JL, McGuire JL, Hadly EA. Small mammal diversity loss in response to late-Pleistocene climatic change. Nature. 2010;465 (7299):771–U775. 10.1038/nature09077 20495547

[60] Hadly EA, Spaeth PA, Li C (2009). Niche conservatism above the species level. Proc Natl Acad Sci.

[61] Marcy AE, Hadly EA, Sherratt E, Garland K, Weisbecker V (2016) Data from: Getting a head in hard soils: Convergent skull evolution and divergent allometric patterns explain shape variation in a highly diverse genus of pocket gophers (*Thomomys*). Dryad Digital Repository. 2016; doi:10.5061/dryad.bj7n9 10.1186/s12862-016-0782-1 PMC5057207 27724858

