# Supplementary material for: Getting a head in hard soils: Convergent skull evolution and divergent allometric patterns explain shape variation in a highly diverse genus of pocket gophers (Thomomys)
Source: BMC Evol Biol. 2016 Oct 10;16:207. doi: 10.1186/s12862-016-0782-1 (PMC5057207; doi:10.1186/s12862-016-0782-1)
Supplement: Additional file 4: — Supplementary methods. (DOCX 79 kb) [file 12862_2016_782_MOESM4_ESM.docx]

Additional File 4: Supplementary Methods

**Protocol for photography:**

Photography settings: two lights, one hot and one soft, on either side of a vertical camera mount with the specimen placed on a black background below. A digital single-lens reflex camera with shutter speed at 1/5 seconds, aperture at f/36, exposure composition at 0 EV, sensitivity at ISO 200, white balance at incandescent, and metering mode at center-weighted was used to take JPEG images with dimensions 3872 by 2592 pixels without zoom, flash, or flexible program.

For the lateral view, crania were placed on the right jugal on modeling clay and against a T-square ruler to ensure a 90-degree angle with the mounting base; posterior positioned towards the hot light. For the ventral view, crania were placed resting on the frontals with posterior towards the hot light and anterior towards the soft light.

For the anterior view, humeri were laid on the support of the trochlea with the distal end towards the hot light. For the lateral view, humeri laid on the support of the deltopectoral crest with distal end towards the hot light.
