# Supplementary figures and images for: Getting a head in hard soils: Convergent skull evolution and divergent allometric patterns explain shape variation in a highly diverse genus of pocket gophers (Thomomys)
Source: BMC Evol Biol. 2016 Oct 10;16:207. doi: 10.1186/s12862-016-0782-1 (PMC5057207; doi:10.1186/s12862-016-0782-1)

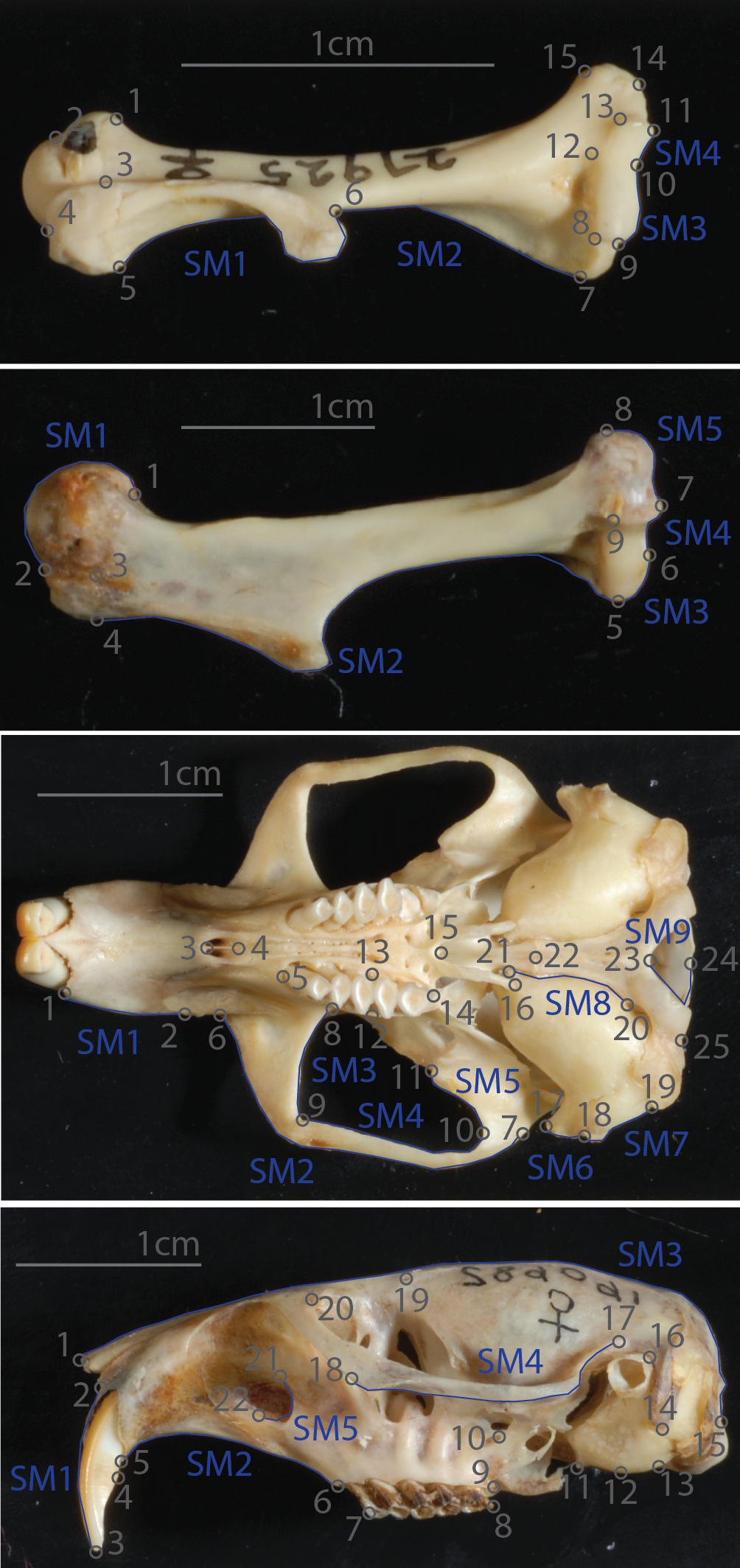

Supplement: Additional file 5: Figure S1. — Landmark and semilandmark key. Red circles indicate location of landmarks, blue lines indicate sliding paths for semilandmarks on lateral cranial view a, ventral cranial view b, anterior humeral view c, and lateral humeral view d. Definitions in Additional file 6: Table S4. (PNG 2510 kb) [file 12862_2016_782_MOESM5_ESM.png]

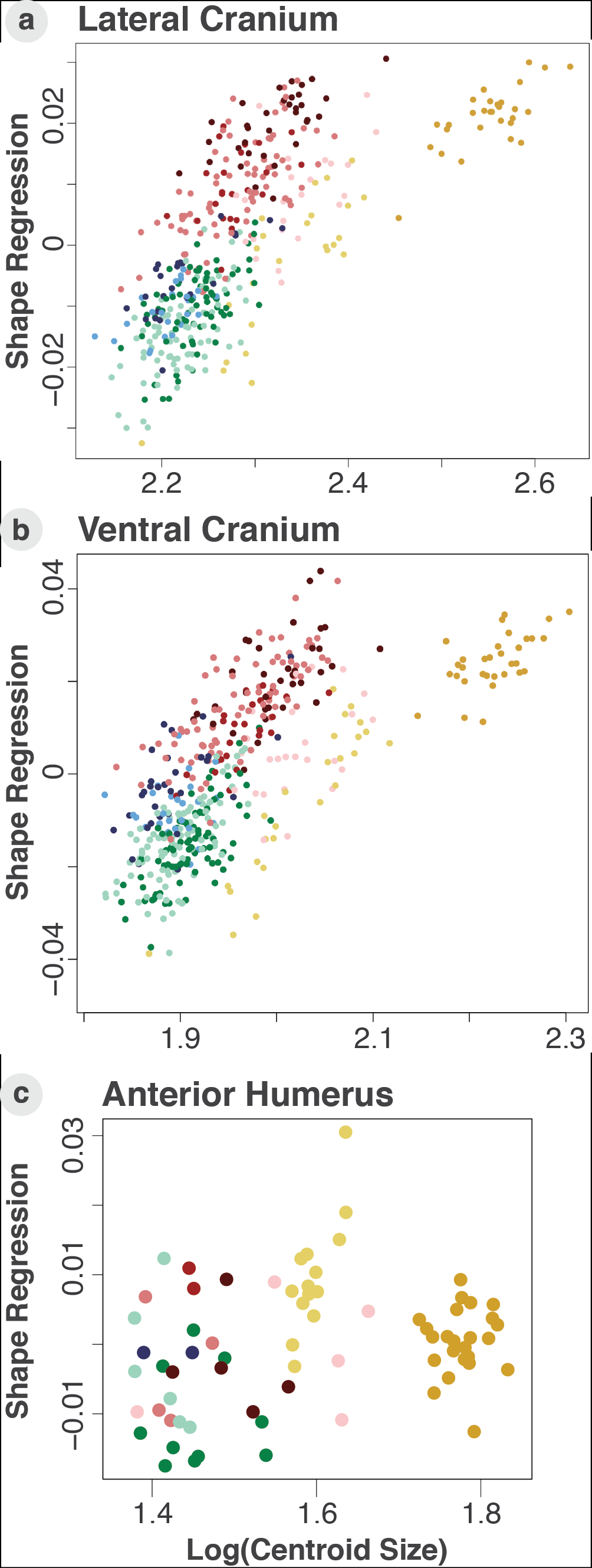

Supplement: Additional file 8: Figure S2. — Multivariate regression plots. The regression score (after [55]) of lateral a and ventral b cranial shape versus log centroid size show the allometric variation across all taxa. These data underlie the predicted shape allometric slopes in Fig. 4b and e, respectively. Regression score plot for humeral data c demonstrates that the T. M. Bottae clade taxa and the subgenus Thomomys taxa have similar centroid sizes. (PNG 274 kb) [file 12862_2016_782_MOESM8_ESM.png]
