# Supplementary material for: Getting a head in hard soils: Convergent skull evolution and divergent allometric patterns explain shape variation in a highly diverse genus of pocket gophers (Thomomys)
Source: BMC Evol Biol. 2016 Oct 10;16:207. doi: 10.1186/s12862-016-0782-1 (PMC5057207; doi:10.1186/s12862-016-0782-1)
Supplement: Additional file 7: — Lateral humeral shape results. (DOCX 75 kb) [file 12862_2016_782_MOESM7_ESM.docx]

Supplementary file 7: Lateral Humeral Shape Results

Procrustes ANOVA of shape and size

| **Lateral Humeri** |  |  |  |  |  |  |
| --- | --- | --- | --- | --- | --- | --- |
| log(size) | 1 | 0.003970 | 0.0039700 | 0.12353 | 12.6381 | **0.001** |
| subspecies | 7 | 0.010226 | 0.0014609 | 0.31821 | 4.6506 | **0.001** |
| log(size):subspecies | 7 | 0.002234 | 0.0003192 | 0.06952 | 1.0161 | 0.196 |
| residuals | 50 | 0.015707 | 0.0003141 |  |  |  |
| total | 65 | 0.032137 |  |  |  |  |

The effect of centroid size (a proxy for body size) on crania and humeral shape within the 10 distinct genus *Thomomys* taxa evaluated by Procrustes AVOVA (details in methods). Degrees of freedom (Df) for each sums of squares (SS) of each term, model residuals, and the total are presented, along with the coefficient of determination (R^2^), and the F ratio and associated P value. Statistical significance of the models was evaluated by permutation using 1000 iterations. Bold indicates p-values less than 0.05.
